# Predictive relevance of PD-L1 expression with pre-existing TILs in gastric cancer

**DOI:** 10.18632/oncotarget.22079

**Published:** 2017-10-26

**Authors:** Xiaoli Ju, Rong Shen, Pan Huang, Jianhua Zhai, Xiaobin Qian, Qiang Wang, Miao Chen

**Affiliations:** ^1^ Department of Pathology, School of Medicine, Jiangsu University, Zhenjiang, Jiangsu 212013, P.R. China; ^2^ Department of Pathology, The Affiliated People’s Hospital of Jiangsu University, Zhenjiang, Jiangsu 212013, P.R. China; ^3^ Institute of Life Sciences, Jiangsu University, Zhenjiang, Jiangsu 212013, P.R. China

**Keywords:** PD-L1, gastric cancer, TILs, prognosis

## Abstract

Expression of programmed cell death receptor ligand 1 (PD-L1) has been shown to be up-regulated in some gastric cancer patients and to correlate with the density of tumour infiltrating lymphocytes (TILs). However, conflicting results have been reported regarding TILs and the expression of PD-L1 as a prognostic marker for gastric cancer. We investigated the correlation of PD-L1 and TILs expression with clinicpathological characteristics in 105 well characterized gastric cancer patients. PD-L1 expression and CD3+ and CD8+ TILs were evaluated by fluorescent multiplex immunohistochemistry (mIHC) analysis. PD-L1 positive staining on tumour cells was observed in 35% cases and 48% cases showed PD-L1 expression on immune cells. Up-regulated PD-L1 expression on tumour cells and immune cells was associated with high density of pre-existing tumour infiltrating CD3+ and CD8+. In additional, more than 70% tumor infiltrating CD3+ cells were CD3+CD8+ cells. More than 60% PD-L1+ immune cells were PD-L1+CD3+CD8+ cells. PD-L1 expression in tumour cells was associated with poor prognosis and high density CD3+ and CD8+ TILs indicated improved overall survival in gastric cancer patients. Increased PD-L1 expression with low density CD3+ and CD8+ TILs had the shortest overall survival. In accordingly, PD-L1 absence with high density CD3+ and CD8+ TILs indicated the best prognosis. Combination of PD-L1 with pre-existing TILs may be more precise than PD-L1 alone for predicting survival in gastric cancer.

## INTRODUCTION

Gastric cancer (GC) is the fourth most common cancer and second leading cause of cancer associated death for men and women worldwide [1, 2]. Although multidisciplinary therapeutic strategies have improved treatment outcomes, the overall prognosis for gastric cancer patients remains poor. Currently, blocking the programmed death 1 (PD-1) and it’s ligand 1 (PD-L1) immune checkpoint signalling to restore anti-tumour immunity has shown unprecedented rates of durable clinical responses in patients, notably in melanoma, renal, lung, prostate and bladder carcinomas [3–6]. Phase Ib studies of immunotherapy for advanced gastric cancer (KEYNOTE-012) are ongoing, with 22% of patients recorded having an overall response by blocking the PD-1/PD-L1 immune checkpoint [7].

PD-1 is present on the surface of active T and B cells, and PD-L1 is expressed on many types of immune cells. Several tumour types express PD-L1, including gastric cancer [8, 9]. The Interaction of PD-1 and PD-L1 suppresses the CD8+ T cells immune response, induces an immunosuppressive microenvironment within the tumour, and allows the tumour to evade immune destruction [10, 11]. Using an IHC approach, PD-L1 expression was detected in 12%–40% of gastric cancer samples, was only weakly detectible in gastric adenomas, and undetectable in normal gastric tissue controls [12, 13]. Several studies have reported a correlation between PD-L1 expression and the prognosis of cancer patients, and that PD-L1 expression is a predictive biomarker for blocking PD-1/PD-L1 treatment response [13–15]. However, the clinical implications of the existence of PD-L1 in tumours and TILs in the tumour microenvironment are still controversial, and the prognostic potential of these factors is unclear.

There are several reports suggesting that PD-L1 expression is associated with poor prognosis of gastric patients [16–18]. Gastric cancer patients with higher intratumoural and stromal CD8+ T cell density also have higher PD-L1 expression, which is associated with shorter progression free and overall survival times [16]. Patients with enhanced expression of FoxP3 and PD-L1 exhibited a lower overall survival rate and a worse prognosis [18]. There are also many reports demonstrating that PD-L1 expression is associated with good prognosis of gastric cancer patients. In gastric cancer cases of western patients, high PD-L1/PD-1 expression was associated with a significantly better patient outcome [13]. High PD-L1 expression and high density of CD3+ T cells in the tumour microenvironment are better prognostic markers in GC 17. GC patients with high density CD8+ and FoxP3+ TILs showed significantly higher overall survival rates than GC patients with low density CD8+ or FoxP3+ cells [19]. With these conflicting results, the true relationship of PD-L1 expression and TILs with patient clinical outcome is yet to be clarified.

We investigated the expression of PD-L1, CD8 and CD3 in gastric tumour specimens and evaluated the relationship of these factors to clinicopathological characteristics and patient survival. These results may serve as a surrogate marker for PD-L1-positive GCs and may help in validating biomarkers to select patients for immune checkpoint treatment strategies.

## RESULTS

### PD-L1 expression in GC (IHC)

**Figure 1:**
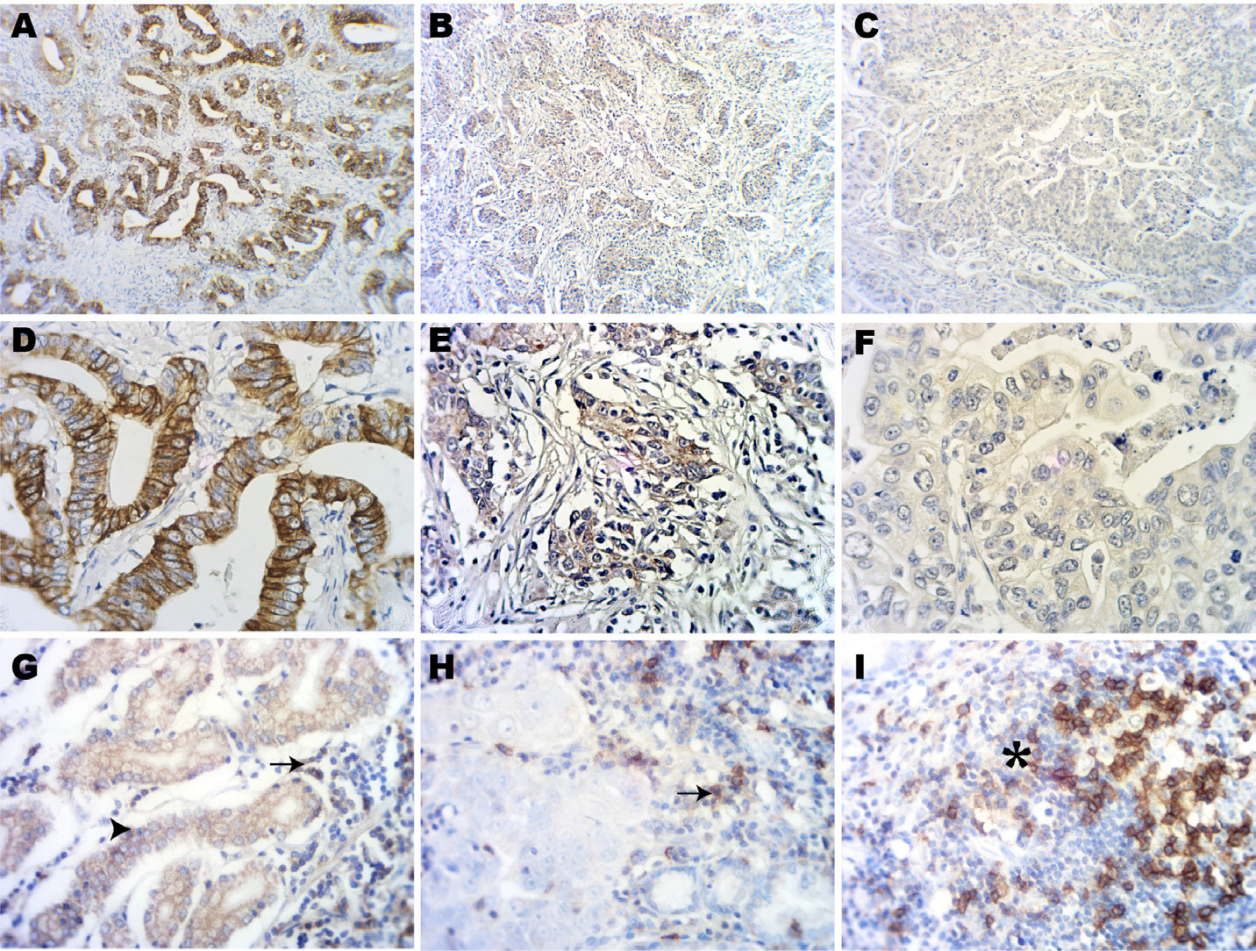
Immunohistochemical staining of PD-L1 in gastric adenocarcinoma tissues (**A**) Strong expression of PD-L1 on tumour cells. (**B**) Weak expression of PD-L1 on tumour cells. (**C**) Negative expression of PD-L1 on tumour cells. (**D**) Strong expression of PD-L1 on tumour cells. (**E**) Weak expression of PD-L1 on tumour cells. (**F**) Negative expression of PD-L1on tumour cells. (**G**) Expression of PD-L1 in tumour cells (arrowhead) and intratumoural immune cells (arrow). (**H**) Expression of PD-L1 on intratumoural immune cells (arrowhead) but not on tumour cells. (**I**) Expression of PD-L1 on intratumoural lymph follicles (star). (A–C original magnification × 100. D–I original magnification × 400).

We examined PD-L1 expression levels in tumour and immune cells in 105 gastric cancer specimens, but not in non-neoplastic gastric epithelium. We observed that PD-L1 was mainly expressed on the membrane and cytoplasm of tumour and immune cells (Figure 1A, 1F). In the present study, 37 of 105 cases (35%) exhibited PD-L1 positive staining (Table 1). Although 90% of the cases showed the same staining intensity within the same sample, different staining intensities were observed between different samples (Figure 1A, 1B, 1D, 1E). Samples with <5% PD-L1 stained tumour cells were considered PD-L1 negative (Figure 1C, 1F) as previous reports [16]. After examining all of the samples, we observed that of PD-L1 positive tumour cell samples, PD-L1 staining in 5% to 65% of tumor cells.

**Table 1:** Clinical, pathological characteristics and their correlation with PD-L1 expression

Characteristic	Total no (*n* = 105)	PD-L1 in tumor cells		*P*-Value	PD-L1 in immune cells		*P*-Value
		Negative	Positive		Negative	Positive	
**Mean age** ± **SD (year)** **Total no**	63.9 ± 9.6 105 (100)	64.1 ± 8.5 68 (65%)	63.2 ± 14.0 37 (35%)	0.970	63.8 ± 13.1 55 (52%)	64.2 ± 8.1 50 (48%)	0.925
**Gender**				0.082			0.051
**Female**	21 (20%)	17 (81%)	4 (19%)		15 (71%)	6 (29%)	
**Male**	84 (80%)	51 (61%)	33 (39%)		40 (48%)	44 (52%)	
**Location**				0.012			0.007
**Proximal**	30 (29%)	25 (83%)	5 (17%)		22 (73%)	8 (27%)	
**Antral**	75 (71%)	43 (57%)	32 (43%)		33 (44%)	42 (56%)	
**AJCC stage**				0.997			0.453
**I**	29 (28%)	19 (66%)	10 (34%)		18 (62%)	11 (38%)	
**II**	33 (31%)	21 (64%)	12 (36%)		17 (52%)	16 (48%)	
**III**	31 (30%)	20 (65%)	11 (35%)		13 (42%)	18 (58%)	
**IV**	12 (11%)	8 (67%)	4 (33%)		7 (58%)	5 (42%)	
**Tumor differentiation**				0.016			1.0
**Poor**	63 (60%)	35 (56%)	28 (44%)		33 (52%)	30 (48%)	
**Moderate to well**	42 (40%)	33 (79%)	9 (21%)		23 (52%)	20 (48%)	
**Tumor growth pattern**				0.183			0.123
**Expansile** **Intermediate** **Infiltrative**	3 (3%) 72 (69%) 30 (29%)	3 (100%) 43 (60%) 22 (73%)	0 (0%) 29 (40%) 8 (27%)		3 (100%) 34 (47%) 18 (60%)	0 (0%) 38 (53%) 12 (40%)	
**Ki67 status** **low** **high** **HER-2 status** **Negative** **Positive**	47 (46%) 58 (54%) 86 (82%) 19 (18%)	36 (77%) 32 (55%) 60 (70%) 8 (42%)	11 (23%) 26 (45%) 26 (30%) 11 (58%)	0.022 0.022	32 (68%) 23 (40%) 48 (56%) 7 (37%)	15 (32%) 35 (60%) 38 (44%) 12 (63%)	0.004 0.134

We also evaluated PD-L1 expression on immune cells. PD-L1 staining in ≥1% of immune cells was considered PD-L1 positive. Fifty of 105 cases (48%) were PD-L1 expression (Table 1). There were 35 cases were PD-L1 positive both on tumour infiltrating immune cells and tumour cells (Figure 1G) and 15 cases PD-L1 expressed on immune cells but not on tumour cells (Figure 1H). PD-L1 positive immune cells were present on intratumoral lymph follicles immune cells (Figure 1I). Of the PD-L1 positive sample, PD-L1 staining was present in 1% to 50% of the immune cells.

### Correlation between PD-L1 expression and clinicopathological features

Clinicopathological features and molecular characteristics according to PD-L1 expression on tumour cells and immune cells are summarized in Table 1.

The study cohort (*n =* 105) included 84 males (80%) and 21 females (20%). According to age, patients were classified into <65y (49%) and ≥65y (51%) subsets. Expression of PD-L1 in tumour cells was associated with location (*P =* 0.012), tumour differentiation (*P =* 0.016), Ki67 status (*P =* 0.022) and HER-2 status (*P =* 0.022). In 30 cases (29%), tumours were located in the gastric cardia and body (17% had PD-L1 positive tumour cells). In 75 cases (71%), tumours were located in the gastric antrum (43% had PD-L1 positive tumour cells). Forty-two cases were moderate to well differentiated (21% had PD-L1 positive tumour cells), and 63 cases were poor differentiated (44% had PD-L1 positive tumour cells). Expression of PD-L1 in tumour cells was significantly associated with the high Ki67 and HER-2 positive cases. In Ki67 high status cases approximately 45% had PD-L1 positive tumour cells. However, in Ki67 low subsets 23% had PD-L1expression in tumour cells. In HER-2 positive cases approximately 58% had PD-L1 positive tumour cells. However, in HER-2 negative subsets less than 30% had PD-L1 positive tumour. Expression of PD-L1 in immune cells was also associated with antral location (*P =* 0.007) and high Ki67 subtype (*P =* 0.004). Interestingly, in Ki67 high cases approximately 60% had PD-L1 positive immune cells. However, in Ki67 low cases 32% had PD-L1 positive immune cells. Unlike in the tumour cells, expression of PD-L1 in immune cells was not significantly associated with the poor differentiation or HER-2 status.

Staging was classified according to the tumour-node-metastasis (TNM) classification of the American Joint Committee on Cancer (AJCC, 7th edition). PD-L1 expression was not significantly associated with age, gender, disease stage or tumour growth pattern on either tumour cells or on immune cells.

### Correlation between PD-L1 and TILs (mIHC)

**Figure 2:**
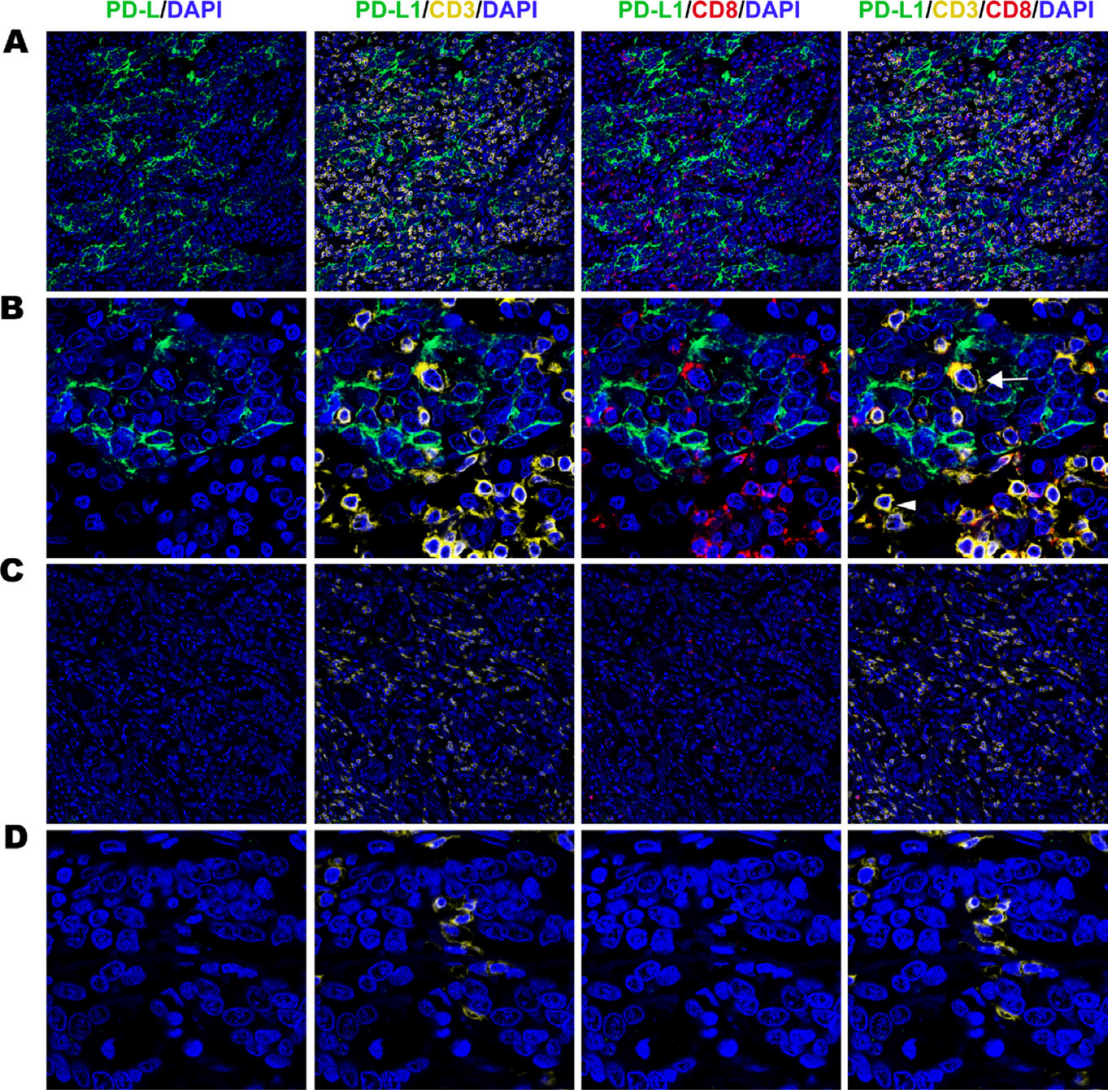
Fluorescent multiplex immunohistochemistry (mIHC) staining pattern for tumour cell PD-L1 and TILs in gastric adenocarcinoma tissues (**A**) Strong expression of PD-L1 on tumour cells with high density of tumour infiltrating CD3+ and CD8+ cells (original magnification × 100). (**B**) Strong expression of PD-L1 on tumour cells with high density of tumour infiltrating CD3+ cells (white arrow) and CD3+CD8+ cells (white arrowhead) (original magnification × 400). (**C**) Negative expression PD-L1 on tumour cells with low density of tumour infiltrating CD3+ and CD8+ cells (original magnification × 100). (**D**) Negative expression PD-L1 on tumour cells with low density of tumour infiltrating CD3+ and CD8+ cells (original magnification × 400).

**Figure 3:**
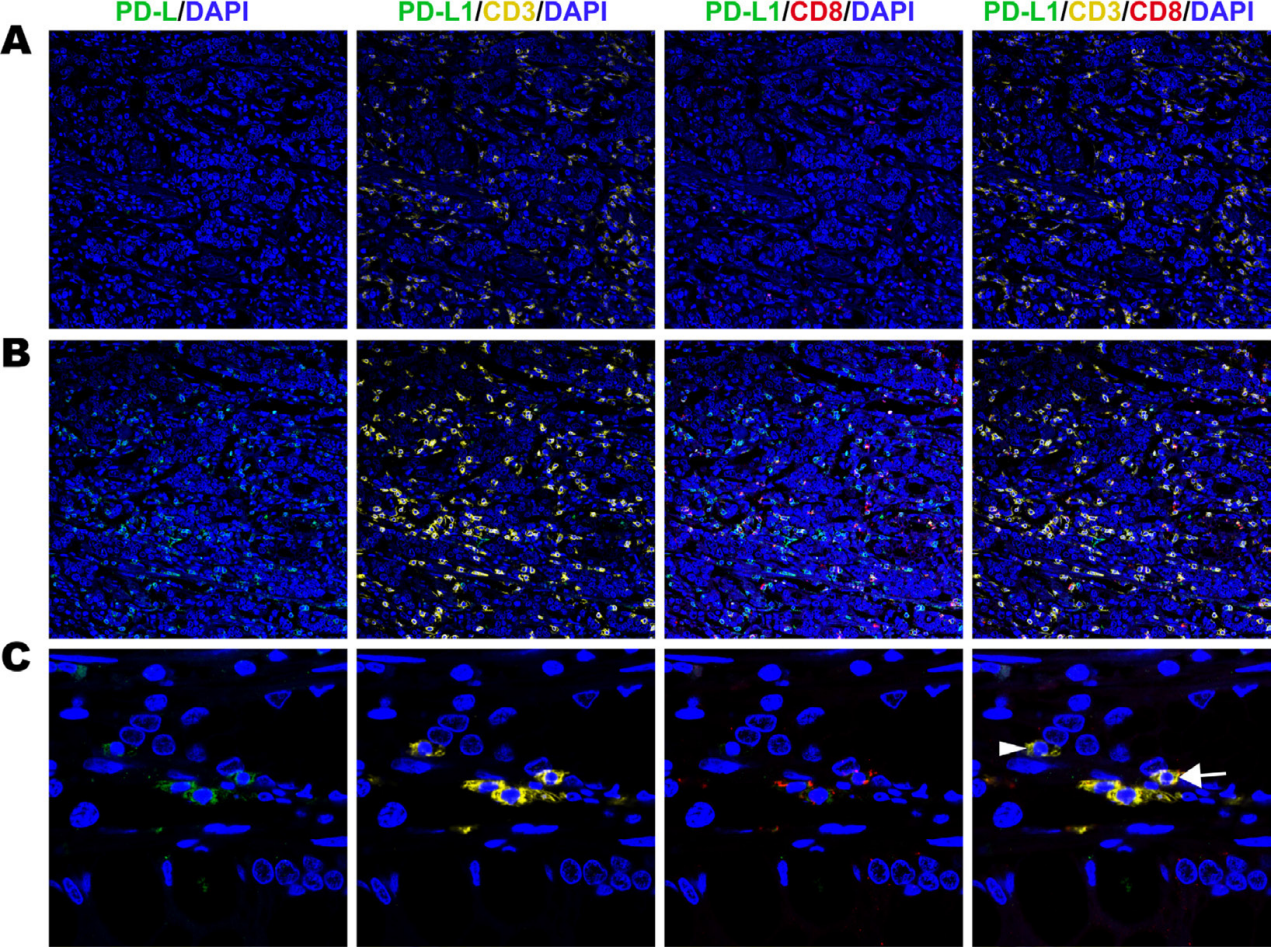
Fluorescent multiplex immunohistochemistry (mIHC) staining pattern for immune cell PD-L1 and TILs in gastric adenocarcinoma tissues (**A**) Negative expression of PD-L1 on immnune cells with low density of tumour infiltrating CD3+ and CD8+ cells (original magnification × 100). (**B**) Positive expression of PD-L1 on immune cells with high density of tumour infiltrating CD3+ cells and CD8+ cells (original magnification ×100). (**C**) Positive expression PD-L1 on tumour cells with low density of tumour infiltrating PD-L1+CD3+ (white arrow) and PD-L1+CD3+CD8+ cells (white arrowhead) (original magnification × 400).

Increased PD-L1 expression on tumor cells and immune cells both positively correlated with CD3+ and CD8+ cell infiltration in gastric cancer. PD-L1 positive in tumour cell subset had a high density of tumor infiltrating CD3+ cells and CD8+ cells (Figure 2A). And more than 70% CD3+ cells were CD3+ CD8+ cells (Figure 2B). PD-L1 negative in tumour cell subset had a low density of tumor infiltrating CD3+ cells and CD8+ cells (Figure 2C). Especially the CD3+CD8+ cells was much fewer in PD-L1 negative subset (Figure 2D). In the PD-L1 positive immune cell subset similar results were observed. PD-L1 negative immune cell cases were infiltrated with low density of CD3+ TILs and CD8+ TILs (Figure 3A). High density of CD3+ TILs and CD8+ TILs were observed in PD-L1 positive immune cell cases (Figure 3B). Approximately 80% PD-L1+ immune cells were PD-L1+ CD3+ and 60% were PD-L1+ CD3+CD8+ (Figure 3C).

Tumor infiltrating CD3+ and CD8+ cells were counted, scored as 1 (<1%), 2 (1–9%), 3 (10–20%), or 4 (>20%) and classified into low and high subsets. The correlation between density of different TIL types and PD-L1 expression was summarized in Table 2. Approximately 47% had a high density of CD3+ TILs and 46% had a high density of CD8+ TILs (Table 2). In the PD-L1 positive tumour cell subset, a significantly higher proportion of CD3+ TILs and CD8+ TILs were observed. In the PD-L1 positive tumour cell subset, approximately 62% had a high density of CD3+ TILs and CD8+ TILs. In contrast, only 38% of the PD-L1 negative immune cell subset had a high density of CD3+ TILs, 37% had a high density of CD8+ TILs. In the PD-L1 positive immune cell cases, approximately 66% had a high density of CD3+ TIL and CD8+ TILs. In contrast, only 29% of the PD-L1 negative immune cell subset had a high density of CD3+ TILs, 27% had a high density of CD8+ TILs.

**Table 2:** Relationship of PD-L1 expression and T cell density in gastric cancer

Characteristic	CD3		*P*-Value	CD8		*P*-Value
	Low	High		Low	High	
**Total no** **Tumor cells**	56 (53%)	49 (47%)	0.025	57 (54%)	48 (46%)	0.015
**PD-L1 Negative**	42 (62%)	26 (38%)		43 (63%)	25 (37%)	
**PD-L1 Positive**	14 (48%)	23 (62%)		14 (48%)	23 (62%)	
**Immune cells** **PD-L1 Negative** **PD-L1Positive**	39 (71%) 17 (34%)	16 (29%) 33 (66%)	0.001	40 (73%) 17 (34%)	15 (27%) 33 (66%)	0.000

These results demonstrated that PD-L1 expression in tumor cells and immune cells was positively associated with the density of CD3+ and CD8+ TILs.

### Survival analysis based on PD-L1 expression and TILs

**Figure 4:**
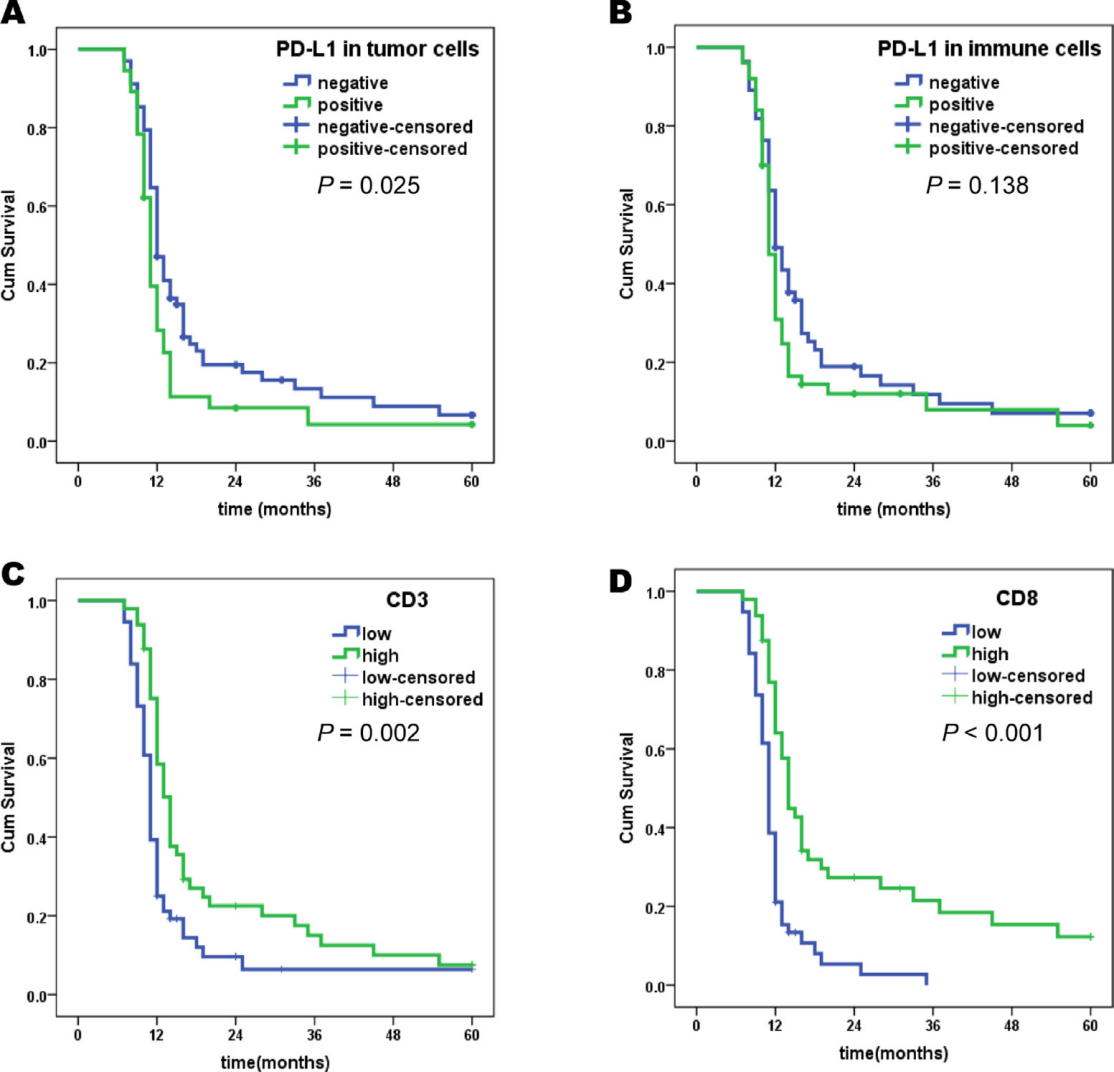
Kaplan-Meier survival analysis with Log-Rank test PD-L1 and TILs (**A**) Survival curves of PD-L1 positive and negative in tumour cells. (**B**) Survival curves of PD-L1 positive and negative in immune cells. (**C**) Survival curves of low-density and high-density CD3+ TILs. (**D**) Survival curves of low-density and high-density CD8+ TILs.

We performed a Kaplan-Meier analysis and Log-Rank test to assess the prognostic role of PD-L1 expression in gastric cancer. PD-L1 expression in tumour cells was associated with worse prognosis. The PD-L1 positive tumour cell subset had a 14.2 month cumulative survival and the PD-L1 negative tumour cell subset had an 18.6 month cumulative survival time (Figure 4A). The difference in cumulative survival between PD-L1 positive and PD-L1 negative subsets in the whole population was significant (*P =* 0.025). There was no significant difference (*P =* 0.138) in cumulative survival time between PD-L1 positive and PD-L1 negative immune cell subsets in the whole population (Figure 4B). Previous reports indicated that the density of TILs was associated with prognosis of cancer patients [10, 16–19]. Significant longer cumulative survival time was observed in high density CD3+ and CD8+ subsets (Figure 4C, 4D).

**Figure 5:**
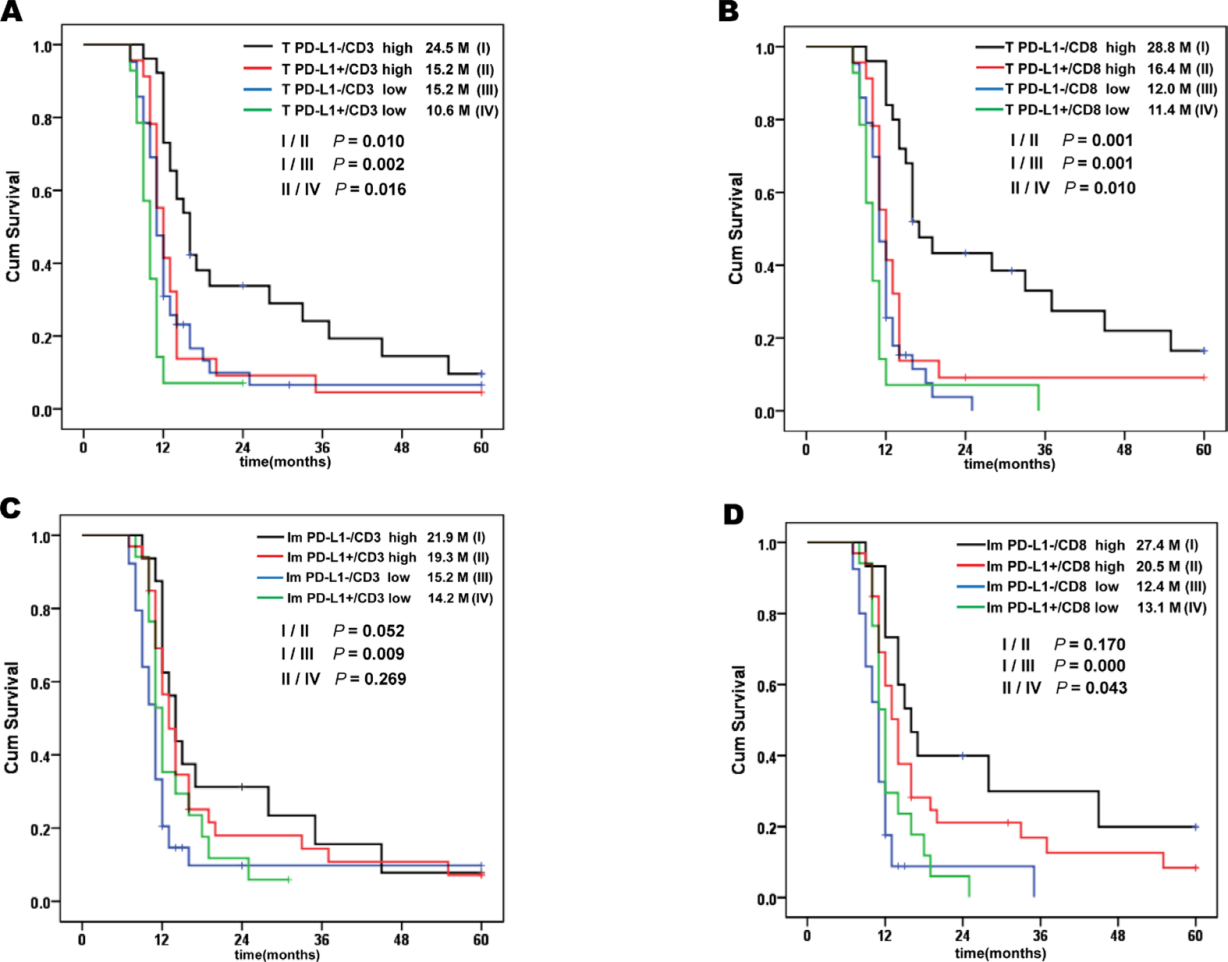
Kaplan-Meier survival analysis with Log-Rank test of PD-L1 combined with TILs (**A**) Survival curves of positive and negative expression of PD-L1 on tumour cells with low density and high density tumour infiltrating CD3+ cells. (**B**) Survival curves of positive and negative expression of PD-L1 on tumour cells with low density and high density tumour infiltrating CD8+ cells. (**C**) Survival curves of positive and negative expression of PD-L1 on immune cells with low density and high density tumour infiltrating CD3+ cells. (**D**) Survival curves of positive and negative expression of PD-L1 on immune cells with low density and high density tumour infiltrating CD8+cells.

We found PD-L1 expression was association with density of CD3+ and CD8+ TILs. PD-L1 expression was combined with TILs density and cumulative survive was analysed. Patients were classified into four groups depend on the PD-L1 expression and density of TILs (Figure 5). We observed the longest cumulative survival time in T PD-L1-/TILs high group. The shortest survival time was observed in T PD-L1+/TILs low group (Figure 5A, 5B). Difference of survival times were significantly. Similarly, PD-L1 expression in immune cells were classified into four groups. The Im PD-L1+/CD3+ low group had the shortest survival time (Figure 5C). However, no significant difference was observed. The Im PD-L1+/CD8+ high group had a significant longer cumulative survival than Im PD-L1+/ CD8+ low group (Figure 5D). Interestingly, Im PD-L1+/CD8+ high group had a longer cumulative survival than Im PD-L1-/CD8+ low group (Figure 5D). However, no significant difference was observed. These results indicated that combination of PD-L1 with pre-excited TILs may be more precise than PD-L1 alone for predicting survival in gastric cancer.

## DISCUSSION

PD-L1 expression on tumour cells has been correlated with tumour cell evasion through down regulation of active T cell mediated immune responses [20, 21]. PD-L1 expression is found in approximately 12% to 46% of gastric cancer patients, suggesting this may be a predictive biomarker for successful PD1/PD-L1 immune check point inhibitor therapy. Expression of PD-L1 has also been shown to be an independent prognostic predictor in gastric cancer.

In this study, we demonstrated that PD-L1 was expressed on tumour cell in 35% of gastric cancer cases. In 48% of gastric cancer cases, PD-L1 was expressed on immune cells. This result was consistent with previous reports. However, even in the PD-L1 positive sample, the overall proportion of PD-L1 stained tumour cells and immune cells was low. We analysed the correlation between PD-L1 expression and several clinicopathologic parameters in gastric cancer. PD-L1 positive cases were found more frequently in tumours located in gastric antrum and in poorly differentiated tumours. No significant associations were found for other parameters, including age, disease stage, and lymph node metastasis. Interestingly, PD-L1 expression in tumour cells correlated significantly with the Ki67 and HER-2 status of gastric cancer. Our data indicated that PD-L1 expression in tumour cells associated with poor-prognostic features such as poor differentiation and high proliferation. This result suggests future investigations of these molecules could be conducive to anti-tumour immunotherapy for gastric cancer.

It has been shown that PD-L1 expression was associated with overall survival in gastric cancer and weak tumour staining of PD-L1 was associated with better overall survival time compared to strong tumour staining of PD-L1 [15–17]. In the present study, we sorted cases into positive and negative subsets according to the percentage of PD-L1 staining cells. We observed that PD-L1 expression on tumour cells was associates with poor-prognostic features, but we did not find a significantly different survival time with or without PD-L1 expression on immune cells. It indicated that PD-L1 expression in tumour cells possibly be an independent predictor biomarkes but not in immune cells.

Previous studies demonstrated that PD-L1 expression strongly correlated with TILs [22]. The density of CD8+ TILs are associated with PD-L1 expression in many type of cancers, such as melanoma [23], human brain metastases, lung cancer [24] and gastric cancer [16]. In colorectal cancer tissue, tumour CD247 (PD-L1) is inversely associated with FoxP3+, but not CD3+, CD8+ or CD45RO+ cell density [25]. Our current study observed PD-L1 expression in tumour cells and immune cells was strongly correlated a high density of CD3+ and CD8+ TILs. And more than 70% CD3+ cells were CD3+ CD8+ double positive cells. Approximately 80% PD-L1+ immune cells were PD-L1+ CD3+ and 60% were PD-L1+ CD3+CD8+ cells. Significantly different survival times were observed between groups of T PD-L1+/TILs high, T PD-L1-/TILs high, T PD-L1+/TILs low and T PD-L1-/TILs low. The T PD-L1+/CD8+ low group had the shortest survival. Accordingly, T PD-L1-/CD8+ high group had the best prognosis. Similar the Im PD-L1+/CD8+ high group had a better outcome than Im PD-L1+/CD8+ low group. Interestingly, Im PD-L1+/CD8+ low group had a longer survival time than PD-L1-/CD8+ low group. Although no significant difference was observed. It suggested that PD-L1 expression in immune cells combined with TILs may have different prognostic value. Larger samples and improved methods may be required for the further studies.

In conclusion, PD-L1 expression on tumour cells and immune cells was positively associated with densities of CD3+ and CD8+ TILs. Up-regulated PD-L1 expression with low density TILs indicated the worst prognosis for gastric cancer patients. Up-regulated PD-L1 expression on tumor cells and immune cells with increased density of CD8+ cells was indicated an improved prognosis than low density of CD8+ TILs. Combination of PD-L1 with pre-excited TILs may be more important than PD-L1 alone for predicting survival in gastric cancer. These findings may have important implications for PD-1/PD-L1 block therapy in gastric cancer patients.

## MATERIALS AND METHODS

### Patients and samples

We collected gastric cancer specimens from 105 patients who were diagnosed and undergoing surgical excision of a primary tumour at the First People’s Hospital of Zhenjiang between January 2009 and June 2011. The characteristics and pathological data of the patients were collected from the pathology department of the First People’s Hospital of Zhenjiang. We excluded patients who received radiotherapy, chemotherapy or other medical intervention before the study. All of the study subjects had completed the follow-up process until July 2016. All the tissue specimens were fixed in formalin and embedded in paraffin for IHC analysis.

### Immunohistochemical analysis

Formalin-fixed and paraffin-embedded samples were obtained from the pathology department of the First People’s Hospital of Zhenjiang. Approximately 4-µm thick sections were cut from the selected samples. The sections were mounted on glass slides and then used for H&E, IHC and mIHC staining.

The diagnosis was verified for all samples using H&E staining. For IHC, sections were deparaffinised, rehydrated and incubated in Antigen retrieval Citra solution, pH 6.0 (BOSTER, Wuhan, China) at 95°C-99°C for 20 min, then cooled to room temperature. Slides were incubated in 3% H_2_O_2_ (BOSTER, Wuhan, China) for 10 min at room temperature to block endogenous enzyme. Then, 5% BSA (BOSTER, Wuhan, China) was used to block unspecific binding for 20 min at room temperature. For IHC, sections were incubated with 1:250 diluted primary antibody PD-L1 (clone: E1L3N, Cell Signaling Technology, MA USA.MA USA) incubated at 4°C over-night. Anti- Rabbit IgG SABC kit (SA1020, BOSTER, Wuhan, China) and DAB detection kit (ZSGB-BIO, Beijing, China) were used. For Fluorescent Multiplex immunohistochemistry (mIHC), firstly, slides were incubated in 1:250 diluted primary antibody CD8 (clone: C8/144B, PD-L1/CD3ε/ CD8α /Multiplex IHC Antibody Panel #65713, Cell Signaling Technology, MA USA.) for 60 minutes at room temperature. After wash, slides were incubated in HRP labeled anti-mouse secondary antibody (SV0001, BOSTER, Wuhan, China) for 30 minutes at room temperature. Wash slides by TNT buffer (0.1 M Tris-HCl, pH 7.5, 0.15M NaCl, 0.05% Tween 20). Sections were keep in dark place and incubated with 1:50 diluted TSA Plus Cyanine 5 (TSA Plus Fluorescence NEL745001KT, PerkineElmer, MA USA) for 5 minutes at room temperature. Slides were stripped in 10mM sodium citrate buffer, pH 6.0 at a sub-boiling temperature for 10 min by microwave. Secondary, 1:250 diluted primary antibody CD3 (clone:D7A6E, PD-L1/CD3ε/CD8α/Multiplex IHC Antibody Panel #65713, Cell Signaling Technology, MA USA.) were add on the sections and incubated in dark for 60 minutes at room temperature. HRP labeled anti-rabbit secondary antibody (SV0002, BOSTER, Wuhan, China) and 1:50 diluted TSA Plus Cyanine 3 (TSA Plus Fluorescence NEL744001KT, PerkineElmer, MA USA) were used. After stripping, slides were incubated with 1:250 diluted PD-L1 (clone: E1L3N, PD-L1/CD3ε/ CD8α /Multiplex IHC Antibody Panel #65713, Cell Signaling Technology, MA USA.) for 60 minutes at room temperature. HRP labeled anti-rabbit secondary antibody (SV0002, BOSTER, Wuhan, China) and 1:50 diluted TSA Plus Fluorescein (TSA Plus Fluorescence NEL741001KT, PerkineElmer, MA USA) were used. Finally, mount sections with coverslips using DAPI (P36941, ProLong^®^ Gold Antifade Mountant with DAPI, Thermo Fisher). Leica Laser confocal microscopy was used for Multi-Color Fluorescence Imaging.

All IHC and mIHC analyses were performed by two independent observers (Shen and Huang) who were unaware of the patients’ record of clinicopathological features. PD-L1 expression in tumour cells was graded into three groups: 0 (no staining), 1 (weak staining), 2 (moderate to intense staining). We counted PD-L1 staining cells per 1000 tumor cells. The percentage of PD-L1 expression tumuor cells was determined based on 10 random areas (HPF 400 × magnification) in each section and the values were average for statistical analysis. Cases in group 1 or 2 with ≥5% PD-L1 in tumour cells were considered positive as previously. Immune cells were scored separately. PD-L1expression immune cells (intratumoral) were counted per 1000 immune cells, and the percentage was determined based on 10 random areas (HPF 400 × magnification) in each section and the values were average for statistical analysis. Cases with ≥1% PD-L1 in immune cells were considered positive. The number and percentage of CD3 and CD8 expression TILs were determined as PD-L1. Expression of CD3 and CD8 in TILs was scored as 1 (<1%), 2 (1–9%), 3 (10–20%), or 4 (>20%). Subsequently, the grades were classified into low (scores 1–2) and high (scores 3–4) subsets. The IHC results for Ki67 and HER-2 expression were obtained from the pathology department of the First People’s Hospital of Zhenjiang.

### Statistical analyses

All statistical analyses were performed using SPSS 22.0 (IBM Corporation, New York, USA). Pearson’s chi-square test and Fisher’s exact test were performed to evaluate the correlation between PD-L1 expression, density of TILs and other clinicopathological features. Kaplan-Meier methods were used to calculate cumulative survival and 95% confidence intervals (CI) were computed. Log-Rank test was used to determine significant difference between survival curves. Student *t*-test was used to analyze the significance of differences between the mean values of two variables. All comparisons were calculated using a two-tailed test*.* Significant differences were considered at *P* < 0.05.
